# Trazodone for management of depression in Parkinson’s disease: expert opinion and proposal for a treatment algorithm

**DOI:** 10.1007/s10072-026-08978-6

**Published:** 2026-05-14

**Authors:** Angelo Antonini, Anna Rita Bentivoglio, Giovanna Calandra-Buonaura, Roberto Ceravolo, Valentina Leta, Maria Teresa Pellecchia, Andrea Pilotto, Alessandra Nicoletti

**Affiliations:** 1 Neurodegenerative Disease Unit, Centre for Rare Neurological Diseases (ERN-RND), Department of Neuroscience, Padua Neuroscience Center (PNC), University of Padova, Padua, Italy; 2 IRCCS San Camillo, Venice, Italy; 3 UOC Neurologia, Fondazione Policlinico Universitario Agostino Gemelli IRCCS-Università Cattolica del Sacro Cuore, Rome, Italy; 4 Department of Biomedical and Neuromotor Sciences, Alma Mater Studiorum – University of Bologna, Bologna, Italy; 5 IRCCS Istituto delle Scienze Neurologiche di Bologna, Bologna, Italy; 6 Centro Clinico Malattie Neurodegenerative Parkinson e Disordini del Movimento, Università di Pisa-Azienda Ospedaliero-Universitaria Pisana, Pisa, Italy; 7 Department of Clinical Neurosciences, Parkinson and Movement Disorders Unit, Fondazione IRCCS Istituto Neurologico “Carlo Besta”, Milan, Italy; 8 Institute of Psychiatry, Psychology and Neuroscience, The Maurice Wohl Clinical Neuroscience Institute, Department of Basic and Clinical Neuroscience, King’s College London, London, UK; 9 Neuroscience Section, Department of Medicine, Surgery and Dentistry “Scuola Medica Salernitana”, University of Salerno, Salerno, Italy; 10 Neurology Unit, Department of continuity of care and frailty ASST, Brescia, Italy; 11 Department of Clinical and Experimental Sciences, University of Brescia, Brescia, Italy; 12 Center for Alzheimer Research, Division of Clinical Geriatrics, Department of Neurobiology, Care Sciences and Society (NVS), Karolinska Institutet, Stockholm, Sweden; 13 Department of Medical, Surgical Sciences and Advanced Technologies G.F. Ingrassia, University of Catania, Catania, Italy

**Keywords:** Trazodone, Depression, Parkinson's disease, Management, Algorithm

## Abstract

**Background:**

Depression is an underrecognized and undertreated condition in Parkinson’s disease (PD). The development of updated, individualized management strategies represents an important unmet need for clinicians and care providers. This review examines the use of trazodone, focusing on its mechanism of action, and proposes a practical algorithm for its use in the management of depression in patients with PD.

**Materials and methods:**

Seven neurologists, experts in movement disorders took part in a structured panel on depression management to exchange ideas and experiences for the implementation of best practice.

**Results:**

The experts agreed that trazodone can be beneficial in PD patients with comorbid depression. The treatment algorithm proposed considers patients in early or more advanced stages of PD. In early stages with mood alterations and anxiety, the suggested treatments are a mood stabilizer, trazodone, or a tricyclic antidepressant. Serotonergic therapy or an SNRI should be considered for patients with low mood but no agitation. In presence of depressive mood with predominant insomnia, high-dose trazodone should be considered along with mirtazapine. In more advanced stages, in the presence of cognitive deficits without agitation low to medium dose trazodone can be considered along with mirtazapine. In case of cognitive deficits with agitation, low to medium dose trazodone is suggested.

**Conclusions:**

The proposed algorithm may assist neurologists in the management of depression in patients with PD by providing specific recommendations on the role of trazodone across different clinical scenarios, allowing for individualized treatment.

## Introduction

Parkinson’s disease (PD) is the second most common progressive neurodegenerative disorder [1, 2], and its prevalence and incidence has consistently increased over the past few decades in most countries [2, 3]. PD severely affects the quality of life due to both motor and non-motor manifestations including mood changes, sleep disturbances, sensory abnormalities, autonomic dysfunctions, and cognitive and behavioral symptoms [4]. PD is thus associated with high burden on patients, caregivers, and healthcare systems [5, 6].

Depression is one of the most frequent non-motor symptoms in all disease stages and occurs in 20–40% of patients depending on the criteria used to assess it and the specific setting [7]. Depression in patients with PD is also associated with high burden on caregivers [8]. It is often under-recognized, underrated and inadequately treated [4, 9]. Moreover, the recognition and management of depression in PD is still a highly debated issue, as its diagnosis is challenging and treatment is therefore managed by psychiatrists rather than neurologists [4].

Depression can be diagnosed using DSM-5 criteria, even though this has limitations in patients with PD [10–12]. In daily practice, it is recommended that all patients with PD undergo screening for depression either by a structured interview or using scales such as the Hamilton Depression Rating Scale (HAM-D), Beck Depression Inventory (BDI), Hospital Anxiety and Depression Scale (HADS), Montgomery-Åsberg Depression Rating Scale (MADRS), and Geriatric Depression Scale (GDS) [7]. While there are no formal guidelines for treating depression in PD, a selective serotonin reuptake inhibitor (SSRI) or a serotonin–norepinephrine reuptake inhibitor (SNRI) are often prescribed while tricyclic antidepressants are generally considered as second-line therapy [7, 13, 14]. Dopaminergic agents have been tested and are efficacious at least on anhedonia, a key component of PD depression [15, 16]. In particular, pramipexole showed superiority over placebo in the largest double-blind study on PD depression [17]. At present, there remains a strong interest in non-pharmacological approaches including cognitive-behavioral therapy [18]. The current approach to treatment is thus tailored to individual health status, severity of depression, comorbidities, and pharmacological regimen in addition to personal preference [13].

Trazodone is an antagonist of a subpopulation of serotonin receptors and serotonin reuptake inhibitor (SARI), and was shown to improve depressive symptoms in patients with PD in a small randomized controlled trial in 2009 [19]. In major depressive disorder, trazodone is well tolerated with a low risk of weight gain and anticholinergic effects as compared to other antidepressants and has shown benefits for control of depressive symptoms [20]. Despite its interesting pharmacological profile, trazodone has not been widely studied clinically as therapy for PD-related depression. Moreover, trazodone has a unique pharmacological profile, is not included in existing algorithms, and may have specific advantages in patients with insomnia, agitation, or cognitive deficits. For these reasons, the present manuscript discusses the use of trazadone, with a focus on its mechanism of action. It also includes a proposal of an algorithm for the use of trazadone in daily practice to further explore its role in PD depression.

## Materials and methods

Seven neurologists, experts in movement disorders and representative of the different regions of the North, Center, and South of Italy, met for a full day in Bologna to discuss the main unmet needs in the management of PD depression and exchanged ideas on implementations for best practice. Emphasis was also placed on optimizing the patient’s journey and establishing a shared therapeutic approach. The insights that emerged were combined into a single flowchart, reviewed and drafted by the chairman, and followed by a unanimous vote for acceptance, which required the approval of all members. Any disagreements were resolved prior to voting through group discussion and subsequent revision.

The present manuscript can be considered a position paper according to the criteria of the European Academy of Neurology [21]. It required the approval of all participating members and was presented in plenary for approval.

### Trazodone: a multimodal mechanism of action

Trazodone has a dual mechanism of action because it inhibits the serotonin transporter like other SSRIs, but also antagonizes type II serotonin receptors, in particular 5-HT_2A_ and 5-HT_2C_ [22, 23]. Trazodone can thus be considered to have a multimodal mechanism of action.

The antidepressant effects of SSRIs and SNRIs are likely due to blockade of the serotonin transporter with serotonin exerting agonistic action on the 5-HT_1A_ receptor [24]. Notwithstanding, the agonistic activity of serotonin also acts on serotonin receptor subtypes, and especially the 5-HT_2A_ and 5-HT_2C_ receptors, which is putatively related to some adverse effects associated with SSRIs and SNRIs, such as anxiety, insomnia, and sexual dysfunction [23]. Unlike SSRIs and SNRIs, however, SARIs such as trazodone also inhibit the serotonin transporter (SERT), increasing the levels of serotonin, while providing 5-HT_2A_ and 5-HT_2C_ receptor antagonism and saturating 5-HT_1_ receptors, thereby minimizing tolerability issues associated with stimulation of 5-HT_2A/2C_ receptors. In addition, it has been hypothesized that simultaneous antagonism of 5-HT_2A/2C_ receptors combined with SERT inhibition may have synergistic effects that potentiate the antidepressant activity of SARIs and thereby improve tolerability [23–27].

**Fig. 1:**
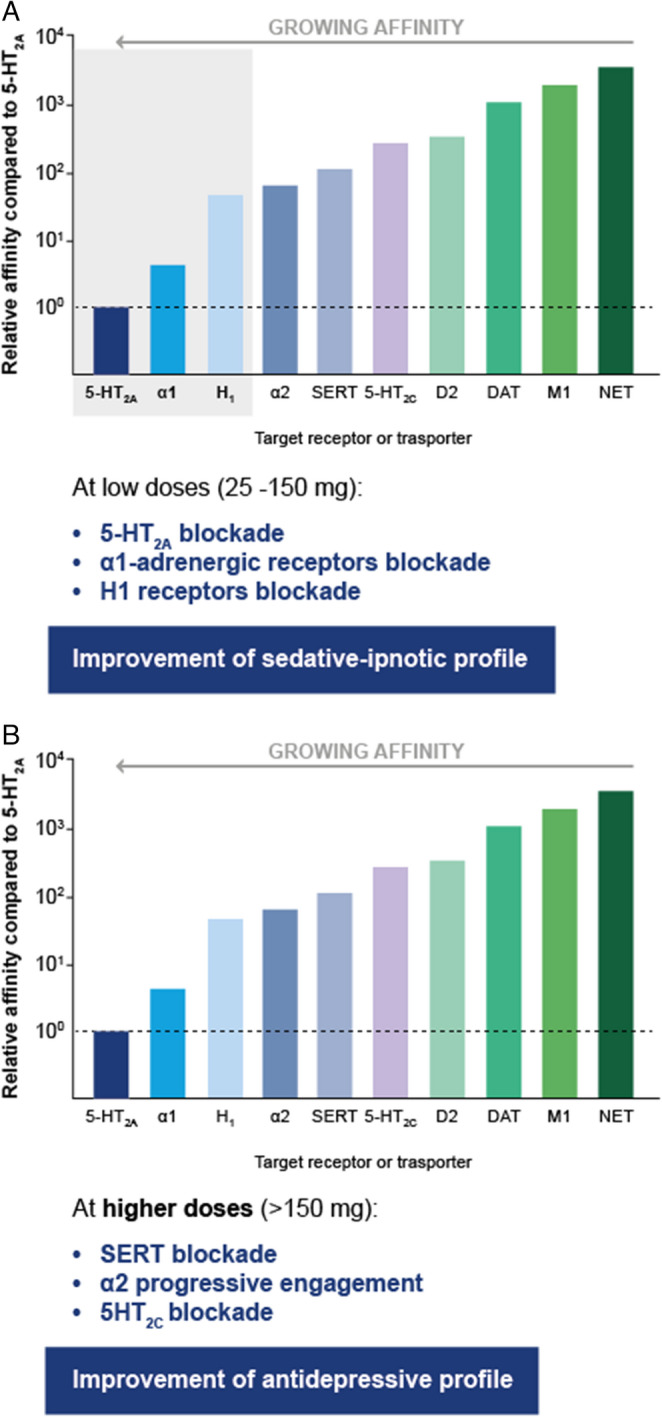
Multifunctional, dose-dependent profile of trazodone [23]. (**A**) Effects at low doses. (**B**) Effects at high doses A

**Fig. 2:**
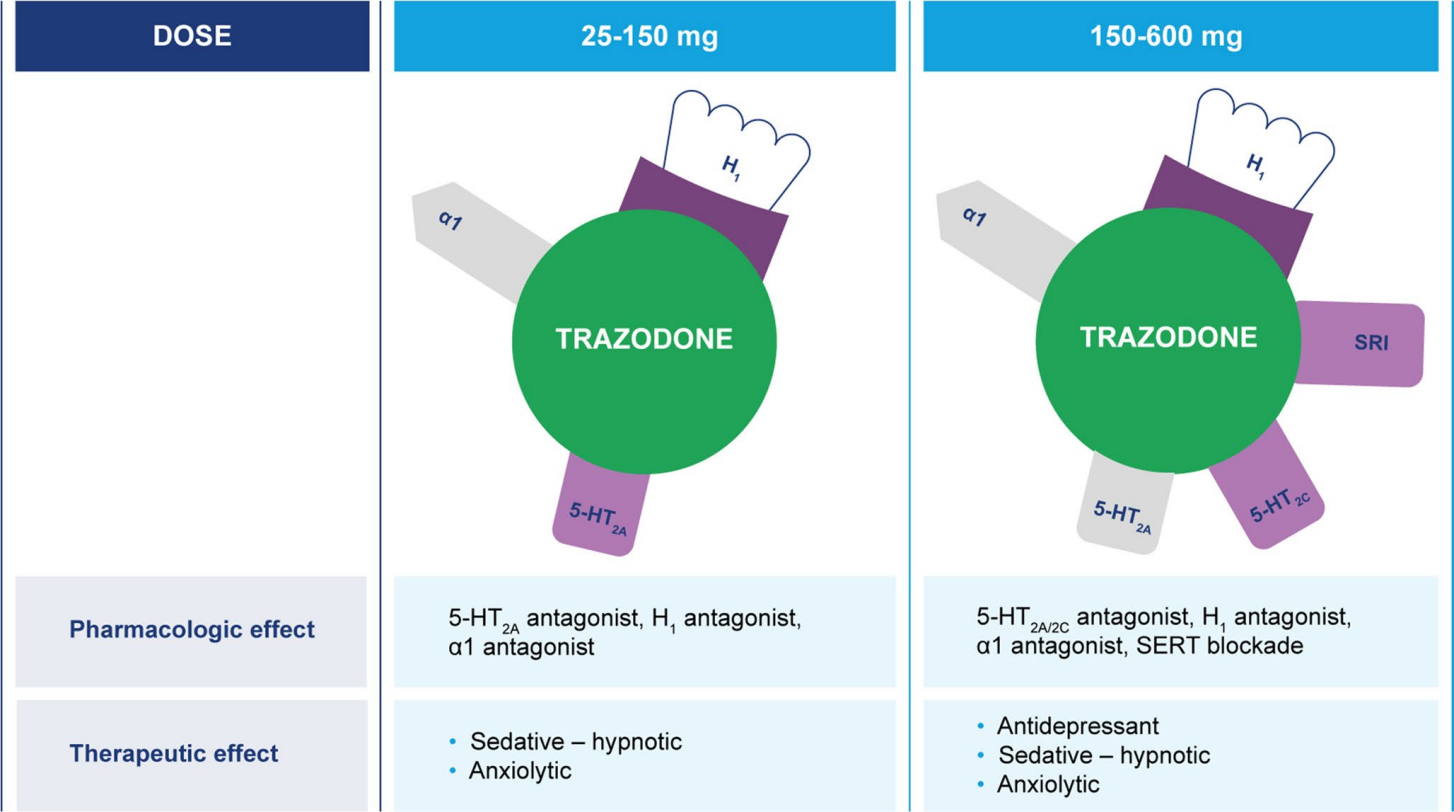
Pharmacodynamic profile and antidepressant effects [31]

Trazodone is a multifunctional agent since it has different actions depending on the dose at which it is administered. In fact, trazodone also antagonizes histamine H1 receptors and α1- and α2-adrenergic receptors, with minimal anticholinergic activity. At low doses of 25–100 mg, trazodone has therapeutic activity as a hypnotic drug (Fig. 1) [23, 28]. Several neurotransmitter systems, including serotonin, dopamine, acetylcholine, noradrenaline, and histamine, are involved in arousal mechanisms [29, 30]. As a consequence, sleep can be induced and arousal can be impaired by inhibiting these neurotransmitter systems. The overall efficacy of trazodone may be rationalized by its ability to inhibit H1 receptors, and the sleep-inducing effect of H1 receptor blockade may be enhanced by simultaneous antagonism of 5-HT_2A_ and α-adrenergic receptors [29, 30]. Thus, at low doses, trazodone blocks receptors involved in the hypnotic effects, such as 5-HT_2A_, α1-adrenergic, and histaminergic H1 receptors [31]. At higher doses (> 150 mg) it inhibits SERT, providing an antidepressant effect as summarized in Fig. 2 [31].

## Formulations of trazodone and implications for treatment

**Fig. 3:**
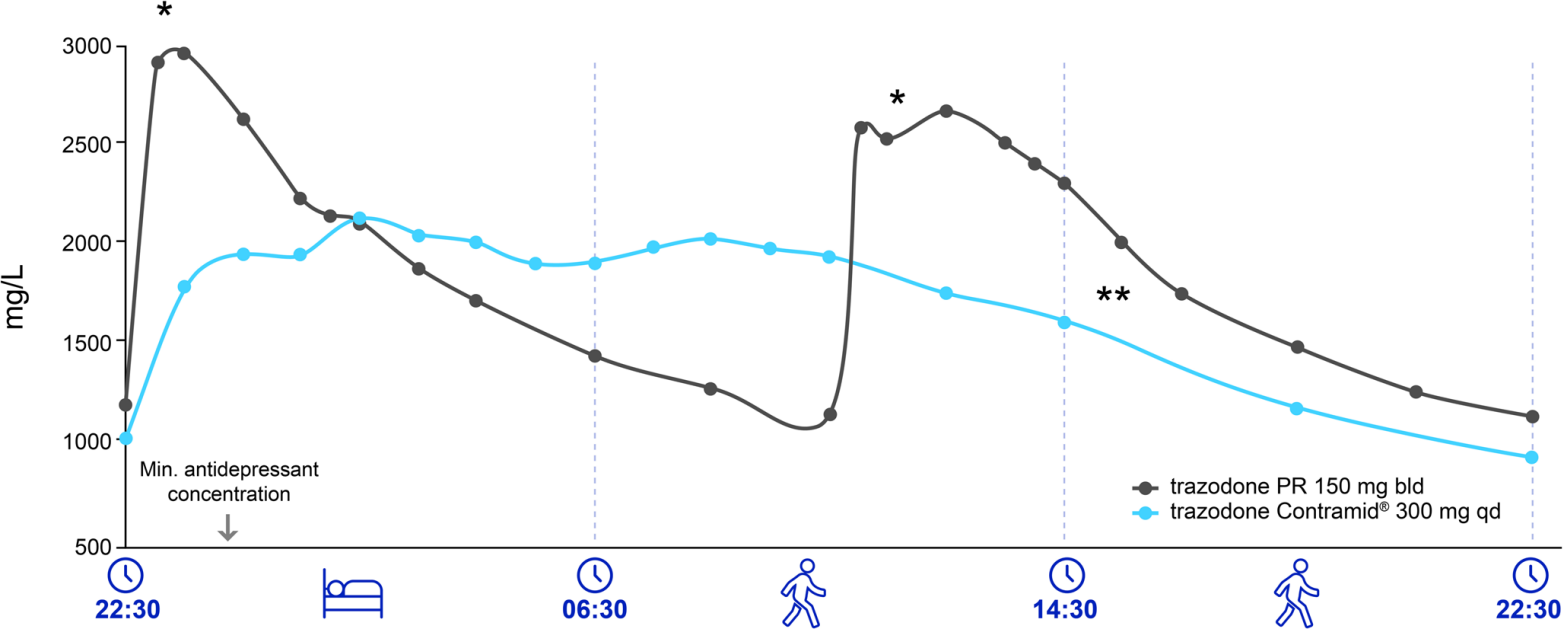
Comparison of the pharmacokinetic properties of the once-daily and prolonged-release formulations of trazodone [32]. *Plasma concentration peaks may be beneficial for patients presenting with more severe symptoms, such as during sleep onset or periods of heightened daytime anxiety and agitation. However, this pharmacokinetic profile can pose challenges for individuals at risk of excessive sedation or when considering long-term treatment strategies**Daytime administration requirements may negatively impact treatment adherence, thereby reducing therapeutic effectiveness. Failure to take or delays in the second dose of trazodone AC can result in plasma concentrations falling below the minimum antidepressant threshold. Sustained adherence is critical to achieving medium- to long-term clinical benefits, attaining remission, and preventing relapse. PR trazodone shows plasma peaks for each administration that could affect drug tolerability, compliance and adherence to the treatment

Depending on the country, trazodone is available in different formulations that can be useful in various clinical scenarios (Fig. 3; Table 1). For example, the prolonged release formulation rapidly reaches maximum blood levels, ensuring rapid effects in patients with more severe daytime anxiety and agitation, while the relatively short elimination half-life of about 6 h reduces the risk of morning drowsiness [20, 33]. The once-daily formulation provides even more gradual and continuous release of the drug, permitting a simple administration schedule, thus improving adherence and tolerability while avoiding highly variable plasma levels [20, 33].

**Table 1:** Characteristics of different formulations of trazodone. Adapted from [32]

Trazodone	Formulations	Cmax	Tmax	t1/2	Food interaction
TZ_IR_	50 mg tablets100 mg tablets25 mg/ml drops 60 mg/ml drops	1.2–1.6 µg/ml	1 h (1.5 h elderly)	6.6 h (9–11 h at steady state)	Slow absorption
TZ_IV/IM_	50 mg/5 ml solution for injection	NA	Immediate	6–8 h	No effect
TZ_PR film-coated_	75 mg tablets 150 mg tablets	0.7–1.2 µg/mL	4 h	12 h	No effect
TZ_COAD_	150 mg tablets 300 mg tablets	~1.5 mg/L (steady state) 2 ± 0.635 µg/mL	7.57 ± 2.3 h	10 h	Increased absorption Administration of TZ_COAD_ 300 mg once daily provides equivalent steady-state exposure to, with a lower C_max_ than, TZ_IR_ 100 mg given 3 times a day. A high-fat meal increase C_max_ but there is no substantial effect on AUC.

## Trazodone: clinical indications and dosage

Trazodone hydrochloride is a potent antidepressant, with anxiety reducing activity [34]. Trazodone is a triazolopyridine derivative chemically unrelated to known tricyclic, tetracyclic and other antidepressant agents [34]. In the EU, trazodone is indicated for depression and depression accompanied by anxiety, as well as for anxiety, in adults. In patients with depression alone or associated with anxiety, trazodone is initially administered at 150 mg/day in two doses after food or as a single dose. The dose for both may be increased up to 300 mg/day in single or split doses. The dose is the same for treatment of depression. For treatment of anxiety, the initial dose is 75 mg/day which can be increased up to 300 mg/day as necessary. Caution should be used when prescribing trazodone to patients with hepatic or renal impairment and dosage adjustment is required. In elderly or frail patients, the recommended initial starting dose is reduced to 100 mg/day given in divided doses or as a single night-time dose, which can be tapered according to clinical needs and side effects. Trazodone is available in different formulations including immediate-release, extended-release tablets and, in some countries, as oral drops.

## Expert opinion on trazodone’s effect on PD non-motor symptoms

The experts agreed that trazodone can be beneficial in PD patients with comorbid depression and cognitive impairment, including hallucinations and sleep disturbances. This may be especially relevant in patients who are not agitated and should be considered before suggesting neuroleptics like quetiapine or clozapine. In patients ≥ 70 years of age with visuospatial cognitive deficits and impaired emotional control, benzodiazepines should be avoided/reduced, since these agents impair attention and executive function [35, 36]. Such patients may benefit from trazodone, which does not affect alertness and attention in the daytime, thereby improving emotional control and preserving daily functioning. Trazodone may also be preferred in presence of sleep disturbance or insomnia with an anxiety-like quality, although attention should be paid to achieving an adequate dose. Indeed, trazodone is commonly prescribed by general neurologists for its hypnotic effect.

In the experts’ opinion, in patients with agitation and insomnia, without evidence of disorganized thinking/jealous delusions, quetiapine may be a valid add-on. On the other hand, in patients with insomnia, frequent and early awakenings not related to nocturia, and non-fluctuating agitated depression, immediate-release trazodone 50–75 mg would be preferred; clozapine should be second-choice and reserved for more resistant cases including dopaminergic psychosis due to the need for blood tests. As mentioned, chronic use of benzodiazepines should be discouraged, while a mood stabilizer such as pregabalin, valproate, and oxcarbazepine (reserving a benzodiazepine for acute emergency situations) should be preferred.

Increasing the trazodone dosage does not decrease its effect on sleep but enhances other clinical effects, such as improving bladder detrusor overactivity, which is a frequent issue in PD [37, 38]. In this regard, caution is needed in elderly patients because trazodone may exert or potentiate anticholinergic activity. Regarding the adverse events of trazodone, there is no evidence of movement disorders. For delirium in the elderly, trazodone has been considered as a candidate for first-line treatment by some authors [39]. Thus, the experts agree that trazodone can be considered in elderly patients with PD and depression since it does not worsen the cognitive profile and may show benefits on other non-motor symptoms such as nocturia.

According to the experts, trazodone may also be considered in PD patients with depressive symptoms and no cognitive decline. Indeed, the use of dopamine agonists (DA) is being increasingly reduced and it may be considered to limit these drugs, in particular in the elderly with PD [40]. In this category of patients, trazodone could be used in lieu of dopamine agonists, although attention is needed when utilizing a dose of > 150 mg/day. In the lack of response, it may be worthwhile to increase the dose before discontinuing it, although in daily practice the experts reported a general apprehension in prescribing higher doses. At present, in clinical practice most neurologists do not consider prescribing trazodone as first choice for depressed PD patients, since there is poor evidence supporting trazodone as an antidepressant in PD. In the experts’ opinion, however, trazodone may be especially useful in different categories of eligible PD patients, as discussed above.

## Proposed algorithm for management of depression in patients with PD

**Fig. 4:**
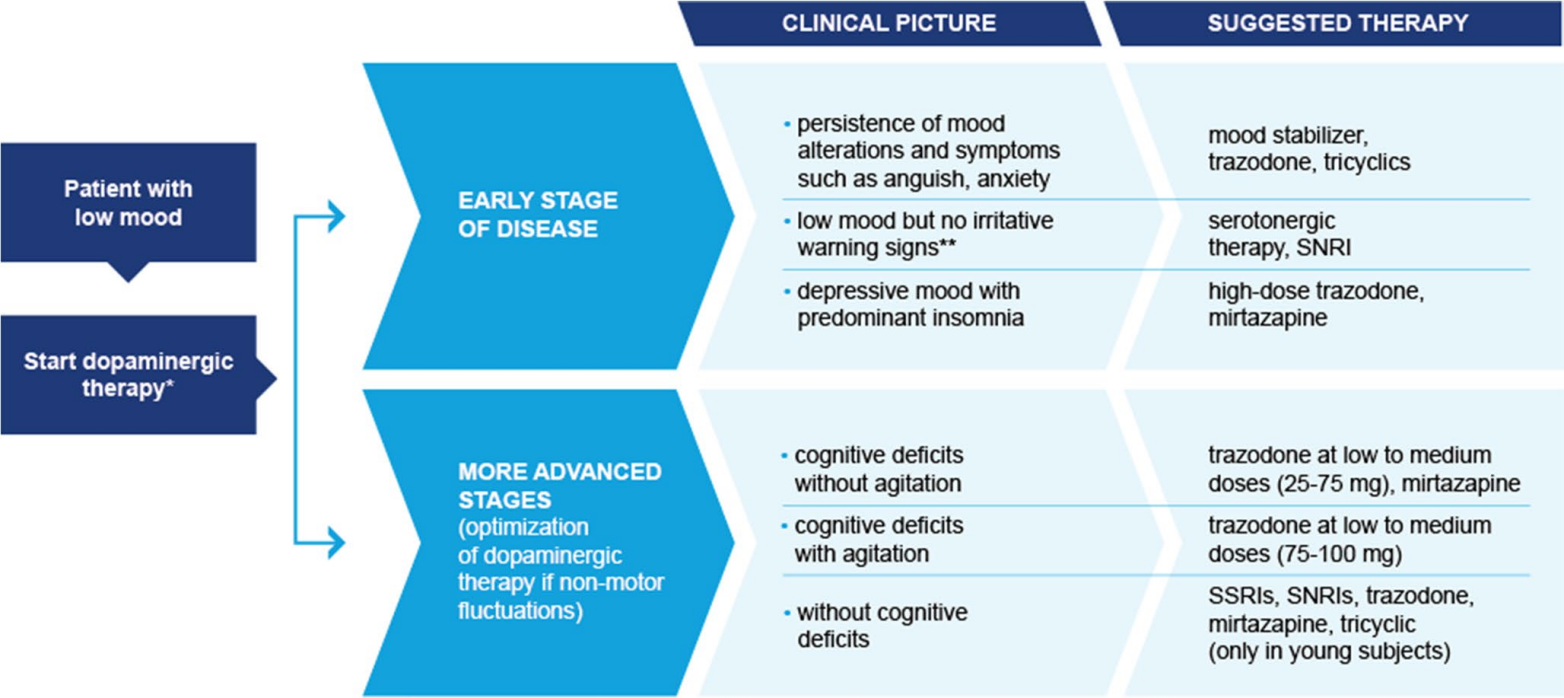
Proposal of an algorithm for management of depression in patients with PD^a^. *pramipexole is the only dopamine agonist with strong evidence for improvement of mood disorders; **patient eligible for trazodone 150–300 mg; ^a^if pain is present: duloxetine or pregabalin and gabapentin

Based on expert consensus and the available clinical evidence, a shared treatment algorithm was proposed for the management of depression in patients with PD (Fig. 4). The algorithm differentiates between early-stage and advanced-stage PD, acknowledging that the clinical expression and neurobiological mechanisms of depression change as the disease progresses. In early stages, depressive symptoms are frequently driven by dysfunction of mesolimbic dopaminergic circuits involved in reward and motivation, whereas in advanced stages the clinical picture is often influenced by widespread neurodegeneration affecting serotonergic, noradrenergic, and cholinergic systems, frequently accompanied by cognitive impairment and sleep disturbances.

### Early stages of Parkinson’s disease

- Predominant anhedonia and motivational deficits.

In the early stages of PD, anhedonia, apathy, and reduced motivation are frequently the predominant components of depressive symptomatology. These features are thought to reflect dysfunction within the mesolimbic dopaminergic pathway, particularly projections from the ventral tegmental area to the nucleus accumbens and prefrontal cortex. Because of this pathophysiological mechanism, dopamine agonists may represent an appropriate therapeutic strategy, particularly in patients whose depressive syndrome is characterized primarily by loss of reward sensitivity rather than pervasive negative mood.

Evidence supporting this approach derives from several clinical trials. In an Italian multicenter parallel-group randomized study comparing pramipexole with sertraline, both treatments produced a reduction in HAM-D scores over 12 weeks. However, the proportion of patients achieving remission (defined as a final HAM-D score ≤ 8) was significantly higher in the pramipexole group. Furthermore, a randomized, double-blind, placebo-controlled trial demonstrated that pramipexole significantly improved depressive symptoms in patients with PD, largely through a direct antidepressant effect independent of motor improvement.

Despite these potential benefits, dopamine agonists must be prescribed with caution due to their association with impulse control disorders, including pathological gambling, compulsive shopping, hypersexuality, and compulsive eating. These behaviors are thought to result from excessive stimulation of the mesolimbic reward system and may significantly impair social functioning and quality of life. Consequently, careful monitoring for behavioral side effects is essential when dopamine agonists are used to treat depressive symptoms in PD.

b) Depression with anxiety or emotional instability

When depressive symptoms are accompanied by prominent anxiety, irritability, or emotional lability, treatment strategies targeting serotonergic and noradrenergic systems may be particularly beneficial. Anxiety is highly prevalent in PD and is believed to reflect alterations in serotonergic projections from the raphe nuclei and noradrenergic pathways originating in the locus coeruleus.

In this clinical context, potential therapeutic options include trazodone, tricyclic antidepressants (TCAs), or mood stabilizing agents. Trazodone may be particularly useful because of its combined antidepressant, anxiolytic, and sedative effects, mediated through serotonin 5-HT_2_ receptor antagonism and histamine H1 blockade. TCAs may also be effective due to their dual inhibition of serotonin and norepinephrine reuptake; however, their use requires careful monitoring because of anticholinergic effects and cardiovascular risks, particularly in older patients.

c) Depression characterized by low mood without agitation

For patients presenting primarily with persistent low mood in the absence of significant anxiety or agitation, serotonergic antidepressants represent a widely accepted first-line pharmacological option. In particular, SSRIs and SNRIs are commonly used because of their favorable safety and tolerability profile.

SSRIs such as sertraline, citalopram, and escitalopram have demonstrated efficacy in treating depressive symptoms in PD in several randomized trials and are generally well tolerated. SNRIs such as venlafaxine may be particularly beneficial in patients with fatigue, reduced energy, or pain symptoms, given their additional noradrenergic activity. In this subgroup of patients, trazodone may also represent a therapeutic option, particularly in the presence of mild sleep disturbances or when a sedating antidepressant is preferred.

d) Depression with predominant insomnia

Sleep disturbances are highly prevalent in PD and often exacerbate depressive symptoms. When insomnia is a prominent feature of the depressive syndrome, treatment strategies should address both mood and sleep regulation. In these cases, experts believe that higher doses of trazodone (typically 150 mg/day or higher) may be considered even if the supporting evidence is lacking in PD. At lower doses (25–100 mg), trazodone exerts mainly sedative effects through histamine H1 and α1-adrenergic receptor blockade, improving sleep initiation and continuity. At higher doses, additional serotonin reuptake inhibition contributes to its antidepressant activity.

Mirtazapine represents another suitable option in patients with depression associated with insomnia. By antagonizing presynaptic α2-adrenergic receptors, mirtazapine enhances both serotonergic and noradrenergic neurotransmission, while its potent antihistaminergic activity promotes sleep. In addition, mirtazapine may improve appetite and reduce anxiety symptoms, which can be beneficial in some patients with PD who experience weight loss or reduced nutritional intake.

e) Advanced stages of Parkinson’s disease

As PD progresses, the clinical presentation of depression often becomes more complex due to widespread neurodegeneration affecting multiple neurotransmitter systems. Patients may develop cognitive impairment, executive dysfunction, and behavioral disturbances, which significantly influence therapeutic decision-making.

f) Advanced PD with cognitive deficits without agitation

In patients with cognitive impairment but without significant agitation, sedating antidepressants may help address both mood symptoms and sleep disturbances. In this context, low to medium doses of trazodone (approximately 50–150 mg/day) may be considered, either as monotherapy or in combination with mirtazapine. These agents have relatively low anticholinergic activity, which is particularly important in patients with cognitive deficits, as drugs with strong anticholinergic properties may worsen confusion and memory impairment.

g) Advanced PD with cognitive deficits and agitation

When cognitive impairment is accompanied by agitation, irritability, or behavioral disturbances, treatment should prioritize agents with calming and sedative properties. In such cases, low to medium doses of trazodone may be particularly helpful due to their behavior-stabilizing and sleep-promoting effects. The sedative properties of trazodone may contribute to improved sleep–wake regulation and reduction of nocturnal agitation.

### Advanced PD without cognitive impairment

In patients with advanced PD but without significant cognitive deficits, a wider range of antidepressant therapies can be considered. These include: SSRIs, SNRIs trazodone, and mirtazapine. The choice among these agents should be guided by the patient’s predominant symptoms, comorbid conditions, and tolerability profile. Tricyclic antidepressants may also be effective in this population and have demonstrated antidepressant efficacy in PD in several clinical studies. However, their use should generally be limited to younger patients, as TCAs carry a higher risk of anticholinergic adverse effects, orthostatic hypotension, cardiac conduction abnormalities, and cognitive impairment, all of which may be particularly problematic in elderly patients with PD.

### Clinical considerations

Overall, the proposed algorithm emphasizes a personalized and symptom-oriented approach to the treatment of depression in PD. Treatment decisions should take into account the following clinical features:

- stage of Parkinson’s disease.
- predominant depressive phenotype (e.g., anhedonia, anxiety, insomnia).
- presence of cognitive impairment or behavioral disturbances.
- potential adverse effects and drug–drug interactions.
- individual patient characteristics such as age and comorbidities.

Nevertheless, several adverse effects require careful consideration in the PD population, which is typically characterized by advanced age, autonomic dysfunction, and polypharmacy. The most common adverse events associated with trazodone include sedation, dizziness, fatigue, headache, dry mouth, nausea, and constipation. Sedation is largely related to histamine H1 receptor antagonism and may be beneficial in patients with insomnia, but excessive daytime somnolence may impair functioning and increase fall risk. Constipation is particularly relevant in PD because gastrointestinal dysmotility and chronic constipation are already highly prevalent due to autonomic dysfunction. The addition of medications that may worsen bowel motility can therefore exacerbate gastrointestinal symptoms and negatively affect quality of life.

Orthostatic hypotension is another important safety consideration. Patients with PD frequently develop neurogenic orthostatic hypotension as a consequence of autonomic nervous system degeneration. Trazodone may further contribute to blood pressure drops through α1-adrenergic receptor antagonism, potentially leading to dizziness, syncope, and an increased risk of falls. This risk may be amplified when trazodone is used concomitantly with other medications commonly prescribed in PD, such as dopamine agonists, levodopa, antihypertensive agents, or diuretics. Initiating treatment at low doses and titrating gradually may help reduce this risk.

Finally, clinicians should consider potential drug–drug interactions in this population. Trazodone is primarily metabolized by CYP3A4, and concomitant use with strong inhibitors or inducers of this enzyme may alter its plasma concentrations. In addition, the risk of serotonergic toxicity should be considered when trazodone is combined with other serotonergic agents, such as SSRIs or SNRIs. Sedation may also be enhanced when trazodone is coadministered with other central nervous system depressants, including benzodiazepines, antipsychotics, or antihistamines, which are frequently used to manage neuropsychiatric symptoms in PD. Careful dose adjustment and monitoring are therefore recommended to ensure safe use in this vulnerable population.

### Future directions

In considering future directions for the use of trazodone for depression in PD patients, the experts acknowledge that it would be useful to carry out an observational, multicenter study to understand routine clinical practice, assessing treatment effectiveness using validated scales for anxiety and depression. Standardized criteria are used to diagnose depression in PD, but unfortunately scales for monitoring anxiety and depression are not commonly used in daily clinical practice. Moreover, a standardized approach to evaluate the effectiveness of treatments on affective components is lacking.

The experts also agreed that neurologists need greater education regarding both the pathophysiology and the clinical manifestations of depression and the pharmacokinetic characteristics of trazodone, and especially its dose-dependent effects. For this purpose, training materials and focused events may be useful to raise awareness about this important topic, particularly in young neurologists. To achieve the full benefits of trazodone on depression, physicians must be made aware of the high-dose approach with trazodone and identify patients who would benefit the most. In addition, there is a lack of real-world evidence on the use of trazodone in patients with PD and depression, which will be fundamental to provide more specific guidance on its use in routine clinical practice. Finally, the experts noted that there is a lack of communication between psychiatrists and neurologists, and that patients with depression and PD may be managed by psychiatrists, at least in the early stages of the disease when motor symptoms are absent or minimal. Enhanced communication between these professionals is needed and a multidisciplinary approach should be favored to organize and provide comprehensive care for both motor and non-motor symptoms [41].

## Conclusions

Depression in PD is often underrecognized and inadequately treated. Trazodone is frequently used in PD because of its combined antidepressant, anxiolytic, and sedative properties, as well as its minimal anticholinergic activity, which makes it preferable to tricyclic antidepressants and other antidepressants in older individuals and in patients with cognitive impairment [42–45]. The proposed algorithm was developed based on expert opinion with the objective to support neurologists in the accurate diagnosis and effective management of depression in patients with PD in routine clinical practice. Given the complex and heterogeneous nature of depression in PD, multidisciplinary management and careful longitudinal monitoring are essential to optimize therapeutic outcomes and minimize treatment-related complications.
